# Agorophobia

**Published:** 1886-07

**Authors:** A. K. Van Horn

**Affiliations:** Yellow Creek, Illinois


A CASE OF AGOROPHOBIA. *By* A. K. VAN HORN, M. D., *Yellow Creek, Illinois.*

Strictly speaking, the above named disease denotes a fear of spaces, but it also includes the kindred mental condition of a strange unaccountable dread, or terror of being alone without being able to give any reason, or explanation of why the dread is felt. While certain phases of the affection seem to point to a purely mental origin, there are also somatic troubles at times, such as sudden weakness, and tremor of the muscles, with strange sensations in various parts of the body.

Agorophobia is not a newly discovered disease, the noted Pascal having suffered from it; but it was first described by Brück, a German, in 1832, under the title "Schwindel Angst." It seems, however, not to have been closely studied, or its symptoms classified until a few years ago. In fact, opportunities for its investigation are infrequent, as the disease is rare. The majority of reported instances occurred in adult men of education, who mostly concealed their infirmity as long as possible, lest they should be declared insane.

In order to add a mite to the history of the affection, I propose to briefly narrate a case at present under my care, which differs from others on record, in that the patient is a woman, not at all educated, nor particularly intelligent.

Mrs. Y——, a farmer's wife, residing eight miles distant, first called on me nearly three years ago, complaining of a horror of being alone in the house, and of certain strange sensations occurring from one to many times daily, that she called "spells of being nervous." She stated that her health was excellent, and every bodily function in good working order. Looking on it as a case of hysteria, I treated her with various tonics—nervines—the bromides, etc., for several months without benefit, till at last disgusted at my want of success, she ceased her visits. This spring she returned and said that for two years she had been treated almost continuously by different physicians, without benefit, and desired me to make another effort for her relief. I instituted a thorough examination, of which the following is a brief report.

Mrs. Y—— is 34 years old, mother of three children, and a good type of the well-to-do class of farmers' wives. One, at least, of her parents, and all her brothers and sisters have died of consumption; but she presents no indications of the disease. Is of large robust frame, weight at age of 18, 200 lbs.; weight at present, 165 lbs. Dark hair and skin, muscles firm, pulse 80, respiration 20, temperature 98¾°. Bowels very slightly constipated, urine natural, menses regular, no vaginal discharge, sleeps well, tongue slightly coated—says her mouth is foul on waking. Had a severe attack of Roseola early in the spring, which left behind a chronic irritation of throat, and a catarrhal condition of stomach. Aside from this, every bodily function seemed in perfect order, and she chiefly complained of abnormal mental sensations—the unutterable terror of being alone—an inexplicable horror of something awful about to happen. So acute is this mental distress that she has more than once assured me she would rush through a lake of fire or plunge into the wildest torrent to escape the torturing fear. When in her husband's presence, or with congenial companions she is measurably at ease; but the mere presence of company is not sufficient, the person must be to her liking. She also complains of "nervous spells," which occur from once to many times daily and lasting from a few minutes to half an hour. These come on irrespective of who may be present, though less frequent in her happier moods. During these attacks strange wave-like sensations flow up the back of the neck, her head seems to jerk, and a trembling extends to the hands and feet. At first there is a sense of chilliness soon followed by a clammy sweat. Palpitation sometimes occurs and there is always a feeling of prostration and impending death.

Her husband informs me that he has been compelled to spend the past three years almost continuously in her presence. That if he leaves the house, even momentarily, for any purpose, she instantly drops whatever she may be engaged in and frantically seeks his presence, and that in all his farm labor she either accompanies him or a woman to her liking must be hired to remain with her. To add to his discomfort marked suicidal impulses occasionally appear in his wife, as they have in others afflicted with the disease. She is not what is termed a *nervous* woman, and has no dread of uncommon natural occurrences, as wind storms, or heavy thunder, nor has she a childish fear of night and darkness. Her mental symptoms are all summed up and condensed into a frantic, undefined horror of some unknown, awful catastrophe happening to her if left alone, and she meditates suicide only that it seems to offer a door of escape from a mental agony that language fails to picture.

After a careful investigation of her case, I am inclined to the opinion that the disease was brought on by excessive labor in the hayfield during an intensely heated term three years ago. The malady began to manifest itself soon after, and it is certain that nothing of the kind had existed before.

On learning that she had been treated almost continuously for three years, and much of the time by physicians of reputation, I confess I saw but little hope of benefiting her.

I said, however, that I would first attempt to relieve her stomach and throat, and if successful would then try to improve her mental condition. In the course of six weeks she was perfectly restored to health; but there was no improvement in her mental state. As I had previously ran over the entire list of tonics—quinine, strychnine, acid phosphate, arsenic, *et hoc genus omne* in her case, I felt it useless to recur to them again. I ordered her to take effervescing bromo-caffein, a dessertspoonful four times daily. Reported one week later, no better.

I then prescribed celerina, from one to two teaspoonfuls four times daily. Improvement rapidly commenced, and still continues. After using it only one week her husband found that he could leave the house without her instantly following his footprints. She has so far used less than one pint of celerina, and is better than she has been for three years. Of course it is too soon to predict a successful result; but the case is reported more on account of its rarity and peculiar features than from any desire to praise a particular remedy.

